# Genome analysis and knowledge-driven variant interpretation with TGex

**DOI:** 10.1186/s12920-019-0647-8

**Published:** 2019-12-30

**Authors:** Dvir Dahary, Yaron Golan, Yaron Mazor, Ofer Zelig, Ruth Barshir, Michal Twik, Tsippi Iny Stein, Guy Rosner, Revital Kariv, Fei Chen, Qiang Zhang, Yiping Shen, Marilyn Safran, Doron Lancet, Simon Fishilevich

**Affiliations:** 1 Clinical Genetics, LifeMap Sciences Inc., Marshfield, MA 02050 USA; 2 Department of Molecular Genetics, Weizmann Institute of Science, Rehovot, Israel; 3 Department of Gastroenterology, Tel-Aviv Sourasky Medical Center, Tel-Aviv, Israel; 4 Faculty of Medicine, Tel Aviv University, Tel-Aviv, Israel; 5 Genetic and Metabolic Central Laboratory, Birth Defect Prevention Research Institute, Maternal and Child Health Hospital, Children’s Hospital of Guangxi Zhuang Autonomous Region, Nanning, 530002 China; 6 Department of Medical Genetics and Molecular Diagnostic Laboratory, Shanghai Children’s Medical Center, Shanghai Jiao Tong University School of Medicine, Shanghai, 200127 China; 7 Department of Neurology, Harvard Medical School, Division of Genetics and Genomics, Boston Children’s Hospital, Boston, MA 02115 USA

**Keywords:** Next generation sequencing analysis, Clinical variant interpretation and classification, Exome sequencing, Whole genome sequencing, Non-coding variants, Biomedical knowledgebase, Rare genetic diseases, Hamartomatous polyposis

## Abstract

**Background:**

The clinical genetics revolution ushers in great opportunities, accompanied by significant challenges. The fundamental mission in clinical genetics is to analyze genomes, and to identify the most relevant genetic variations underlying a patient’s phenotypes and symptoms. The adoption of Whole Genome Sequencing requires novel capacities for interpretation of non-coding variants.

**Results:**

We present TGex, the Translational Genomics expert, a novel genome variation analysis and interpretation platform, with remarkable exome analysis capacities and a pioneering approach of non-coding variants interpretation. TGex’s main strength is combining state-of-the-art variant filtering with knowledge-driven analysis made possible by VarElect, our highly effective gene-phenotype interpretation tool. VarElect leverages the widely used GeneCards knowledgebase, which integrates information from > 150 automatically-mined data sources. Access to such a comprehensive data compendium also facilitates TGex’s broad variant annotation, supporting evidence exploration, and decision making. TGex has an interactive, user-friendly, and easy adaptive interface, ACMG compliance, and an automated reporting system. Beyond comprehensive whole exome sequence capabilities, TGex encompasses innovative non-coding variants interpretation, towards the goal of maximal exploitation of whole genome sequence analyses in the clinical genetics practice. This is enabled by GeneCards’ recently developed GeneHancer, a novel integrative and fully annotated database of human enhancers and promoters. Examining use-cases from a variety of TGex users world-wide, we demonstrate its high diagnostic yields (42% for single exome and 50% for trios in 1500 rare genetic disease cases) and critical actionable genetic findings. The platform’s support for integration with EHR and LIMS through dedicated APIs facilitates automated retrieval of patient data for TGex’s customizable reporting engine, establishing a rapid and cost-effective workflow for an entire range of clinical genetic testing, including rare disorders, cancer predisposition, tumor biopsies and health screening.

**Conclusions:**

TGex is an innovative tool for the annotation, analysis and prioritization of coding and non-coding genomic variants. It provides access to an extensive knowledgebase of genomic annotations, with intuitive and flexible configuration options, allows quick adaptation, and addresses various workflow requirements. It thus simplifies and accelerates variant interpretation in clinical genetics workflows, with remarkable diagnostic yield, as exemplified in the described use cases.

TGex is available at http://tgex.genecards.org/

## Background

Clinical genetics has progressed remarkably in the last decade, moving rapidly from genotyping selected mutations to whole exome sequencing (WES) and whole genome sequencing (WGS) [1–3]. Improvements in technology and analysis capabilities, accompanied by reduced costs, have revolutionized genomics, enabling one to pinpoint relevant genetic variations within millions of variants in sequenced patients. These advances have extraordinary impact on medical care, clinical diagnostics of rare diseases, discovery of novel pathogenic variants and gene-disease relationships, prenatal testing, genetic counseling, prediction of cancer predisposition, pharmacogenomics and personalized medicine [4–7].

The fundamental mission of a clinical genetics platform is to analyze thousands to millions of genetic variants, and to identify the relevant, typically one or two, genetic variations most likely to underlie the patient’s phenotypes and symptoms. The first applications of scaled clinical exome sequencing applied to undiagnosed patients with suspected genetic conditions yielded a molecular diagnosis rate of ~ 25% [8, 9]. More recently, higher yields were described, with only a handful exceeding the 50% barrier [10, 11]; typically the reports are within the modest range of 25–40% [12–14]. Each technological and informatics enhancement offers an opportunity to improve the diagnostic yield, necessitating optimal variant interpretation as a key avenue to pursue.

Clinical adoption of WGS faces many challenges, including cost, speed of delivery and expert time [15], ambiguities and errors in variant calling and annotation [15–17], undiscovered variant- and gene-disease associations, incomplete views of disease associations within databases [15, 18], genetic and phenotypic heterogeneity [15, 18], and the difficulties posed by incidental findings [19]. For optimal diagnosis rates, clinical genetics analysis requires a knowledge-driven analysis platform, based on a comprehensive and regularly updated knowledgebase, and complying with guidelines for reporting recommendations [5, 20, 21].

Systematic re-analysis of un-solved exomes using up-to-date databases was shown to improve the diagnostic yield [15, 22]. The inclusion of newly discovered variant- and gene-disease associations is a key factor in maximizing the diagnosis rate. However, the wealth of relevant biological information, extremely valuable for that purpose, is typically scattered in numerous databases and tools encompassing genomics, bioinformatics, systems biology and systems medicine. Moreover, browsing and extracting the most relevant pieces of data and reaching comprehensive genetic diagnosis poses an overwhelming challenge.

Our widely used GeneCards Suite [23] provides a comprehensive solution. It constitutes a searchable, integrated biomedical knowledgebase, containing comprehensive information on all human genes and diseases. It includes GeneCards, the human gene database, with consolidated gene-centric data from over 150 sources, including genomic, transcriptomic, proteomic, genetic, clinical, and functional information. It also encompasses MalaCards [24], the human disease companion database which integrates more than 60 sources. This knowledgebase represents an extensive network of annotations and mutual relationships, together with the infrastructure needed for rapid biological interpretation of clinical genetics data. The recent augmentation of the knowledgebase to include an extensive collection of functional non-coding regions (non-coding RNA (ncRNA) genes, enhancers and promoters) provides solid grounds for the analysis of typically un-explored out-of-exome variants in WGS [25, 26].

GeneCards is fortified by wide ranging search capabilities, allowing users to enter any Boolean expression with disease-relevant keywords in order to identify the most relevant genes. To cater to variant disease interpretation, the Suite provides VarElect [27], a leading phenotype-based gene prioritization tool [28]. Gene-based prioritization uses broad information to identify and rank likely damaged genes associated with one or more phenotypes, as opposed to simply identifying potentially damaging variants, facilitating the interpretation of novel variants of known disease-genes [29]. The strength of VarElect lies in its capacity to perform automated GeneCards searches on a long list of Next Generation Sequencing (NGS) candidate variant-containing genes and output a scored, prioritized gene list according to disease phenotype and symptom relationships, using the comprehensive GeneCards information. VarElect not only scores and ranks the genes, but also provides detailed evidence of the associations across sections of molecular and genetic data, which is critical for reviewing results and selecting relevant genes and candidate variants. These features of VarElect are indispensable for prioritization in analyses of the millions of variants detected by WGS.

Variant interpretation tools are evolving from simple command-line-based programs and expert excel-sheet-based reviews to interactive, web-based decision support frameworks. In such platforms, variant and gene prioritization are only one component of a dynamic, multifactorial approach to discovery and diagnosis [29]. In this paper, we describe our recently established TGex (Translational Genomics expert), the GeneCards Suite knowledge-driven clinical genetics analysis platform. TGex combines VarElect’s strength with comprehensive variant annotation and filtering capabilities, within a consolidated user interface that supports browsing, viewing, filtering and interpretation interactively, facilitating review and examination by the genetic analyst. The reporting system of TGex leverages the capabilities of VarElect and the vast amount of structured data available in GeneCards to automatically generate full and comprehensive clinical reports. TGex effectively enables biomedical professionals and scientists, without any prerequisite of bioinformatics skills, to perform genome analysis, all the way from raw patient genetic data in VCF (Variant Call Format) files to detailed reports. TGex’s key innovation and strength is the combination of a comprehensive biomedical knowledgebase with broad variant annotation and gene-phenotype prioritization, and a powerful, interactive, user friendly, and adaptable interface, allowing evidence exploration, decision making, and automatic reporting.

## Implementation

**Fig. 1:**
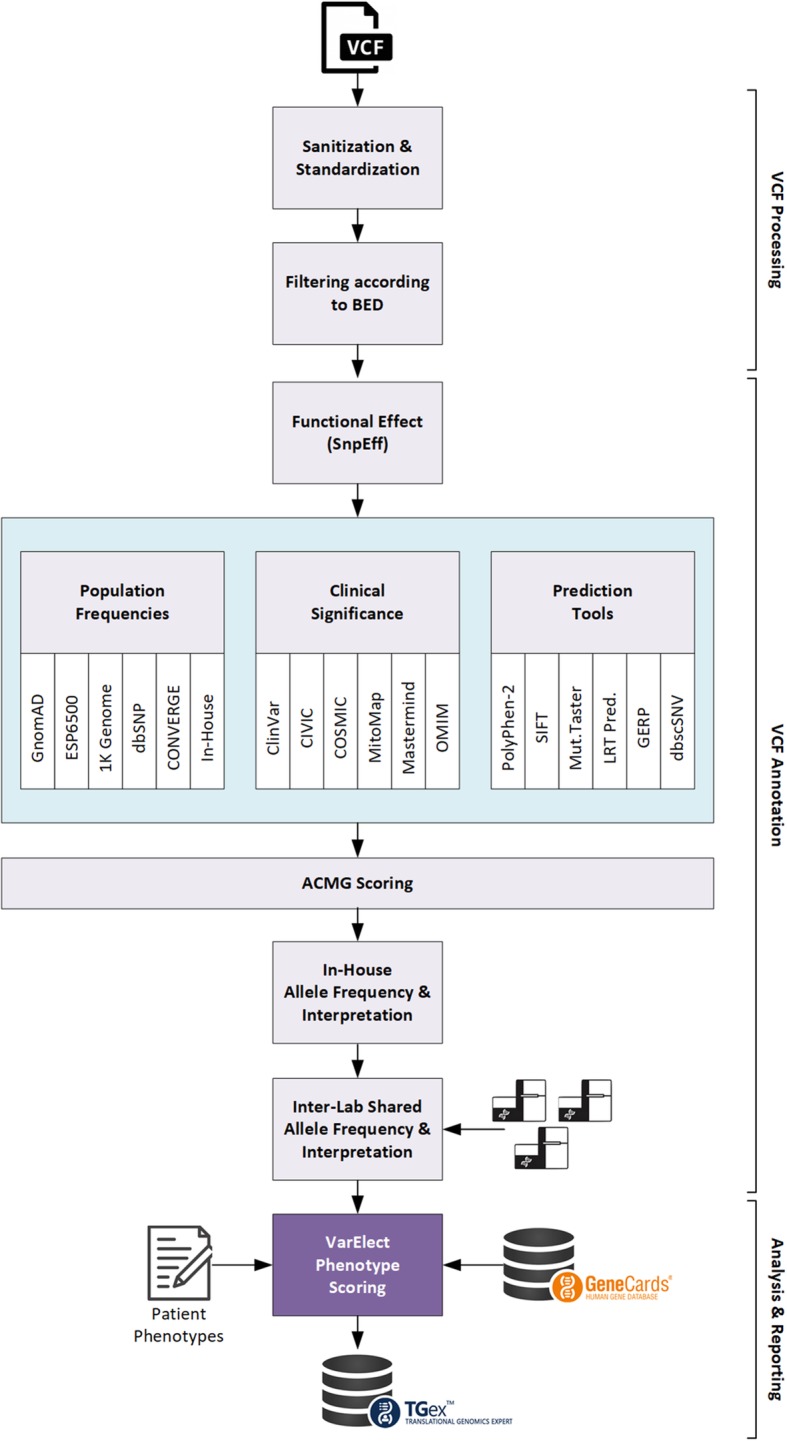
Architecture of variant interpretation in TGex

The general workflow of genetic labs handling sequencing-based genetic tests typically starts from processing and annotating variant files (usually VCF, including all of the variant calls of a certain sample), followed by clinical genetic analysis, and ends with generating a report summarizing the relevant findings. TGex is a clinical genetics analysis platform, providing an end-to-end solution for genetic labs as illustrated in Fig. 1. TGex supports virtually all of the VCF file formats generated by the variety of sequencing machines and primary analysis pipelines found in genetic labs and clinical genetics centers. In addition, TGex accepts patient metadata, sample information (details in Additional file 1: Fig. S1), and clinical details for incorporation in its reporting system. After analysis and interpretation, TGex outputs a report file (PDF or Word) together with a detailed variant annotation file (Excel).

### TGex annotation process

Once a VCF file is uploaded as part of a new analysis in TGex (Fig. 1), it undergoes initial processing, including: (i) ensuring the inclusion of the required fields (validating the VCF format specifications; adding missing VCF headers, e.g. “DP”, “DP4”); (ii) cleaning irrelevant information (e.g. filtering out non-canonical chromosomes; extracting only raw INFO fields if the VCF file is already annotated); (iii) filtering according to relevant genomic regions (using a selected BED file, Exome by default). Following the processing of the VCF file, TGex launches its annotation engine to enrich the VCF with specific information regarding each variant. The first step in the annotation is the prediction of the functional effect with SnpEff [30]. This is followed by applying SnpSift [31] and BCFtools [32] to extract detailed information from dozens of data sources as summarized in Table 1, as well as using proprietary tools developed by LifeMap Sciences (LMS) to split multi-allelic variants and merge identical variants on different transcripts according to the ACMG interpretation guidelines. The next stage is the clinical interpretation of each variant according to the ACMG/AMP 2015 guidelines [51] using all of the available data, including the allele frequency in control populations, clinical significance as provided by specified databases and prediction of the effect of the variant on protein function and transcript integrity using a variety of well-established prediction and scoring tools (detailed in Table 1). Next, TGex uses the data accumulated in the user account (or in a group of accounts sharing information, if applicable) to add in-house information per variant, including in-house allele frequency and user interpretation on variant and gene levels. Finally, additional custom annotations may also be added, including local population-specific frequency data and previous variant interpretations.

**Table 1:** Summary of annotation databases and tools used in TGex

Data Source	Category	Reference
SnpEff	Functional Effect	[30]
ExAC (including GnomAD)	Frequency	[33]
ESP6500	Frequency	[34]
1000 Genomes Project	Frequency	[35]
dbSNP	Frequency	[36]
CONVERGE	Frequency	[37]
ClinVar	Evidence and clinical significance	[38]
CiVIC	Evidence and clinical significance	[39]
COSMIC	Evidence and clinical significance	[40]
MitoMap	Evidence and clinical significance	[41]
Mastermind	Evidence and clinical significance	[42]
OMIM	Evidence and clinical significance	[43]
PolyPhen-2	Effect and Prediction	[44]
SIFT	Effect and Prediction	[45]
MutationTaster	Effect and Prediction	[46]
LRT Prediction	Effect and Prediction	[47]
GERP	Effect and Prediction	[48]
dbscSNV	Effect and Prediction	[49]
RepeatMasker	Genomic repeats	[50]

### Gene-phenotype interpretation

For gene-phenotype prioritization, TGex leverages VarElect, the GeneCards Suite gene phenotyper [27]. VarElect is fully integrated within the TGex analysis screen and reporting system, via the VarElect application programming interface (API). The API input includes user-defined free-text keywords submitted to the GeneCards Knowledgebase search engine (this might include but is not limited to disease names or symptoms in any nomenclature (e.g. HPO [52], UMLS [53] terms)), along with a list of variant-containing genes from the TGex analysis screen. The API output is embedded within the analysis screen, including the gene-keyword score, and the “MiniCards” evidence showing the context of the hits. The “MiniCards” are automatically incorporated in TGex reports, and include extensive gene-phenotype evidence, with dedicated hyperlinks to source databases.

### Versions and data updates

Each report generated in TGex includes documentation of the specific version of the knowledgebase and its annotation databases, which enables tracking, traceability and reproducibility. TGex and the GeneCards knowledgebase are frequently updated with the newest version of dozens of relevant data sources. Knowledgebase updates ensure that the analysis is performed using up-to-date biological knowledge, and often include new annotation sources and new system features. Having a frequently and regularly updated knowledgebase is the basis for our planned reanalysis feature. The genetic and clinical information of each case is stored throughout the lifetime of the TGex account, enabling data querying and case re-analysis using updated knowledgebase versions. This will enable automatic case re-analysis which will trigger sending alerts for outstanding novel findings.

### Automation and APIs

TGex supports customizable, template-based reports with multiple export formats, including Excel, PDF, Word, HTML and JSON. TGex also supports JSON based exports of report data to external reporting engines, which are implemented on-site. The downloading of reports is also available via APIs, enabling laboratory information management systems (LIMS) and electronic health record (EHR) systems to automatically access reports from TGex. The TGex API allows integrators to optimally control the interaction with TGex within broader use-case contexts, including: (i) Integration with primary and secondary analysis pipelines, allowing automated upload and annotation of VCF files; and (ii) Integration with LIMS or EHR systems, by enabling the creation of automated analyses of patient clinical information, and the streamlining of reports from TGex to the LIMS/EHR. TGex also supports fully automated analyses, enabling sophisticated screening protocols to be implemented easily by private and hospital labs. These may include pharmacogenomics, cancer and carrier screening, and newborn screening, among others.

### Data protection compliances

TGex is HIPAA (Health Insurance Portability and Accountability Act) and GDPR (General Data Protection Regulation) compliant.

### Software implementation

TGex is and can be deployed on the cloud (Azure, AliCloud, Amazon and others) or on-premises behind an organization’s firewall. Today, two public cloud-based solutions are offered:

(1) In the Microsoft Azure East US server farm, serving all territories but China, and

(2) In the 21ViaNet Azure Server Farm in Shanghai, serving Chinese users.

The platform consists of four main components:

***TGex Web server*** – Based on ASP.NET and utilizing an MS SQL Server and an Elastic Search server. The TGex server component serves both the TGex web client and other API-based automation clients.

***TGex Web Client*** – A web-based client written in Angular, a popular JavaScript framework. The web client provides the user interface for management of samples, analyses and report lifecycles.

***TGex Annotation server*** – Based on .NET, this server manages the annotation of variant data (in VCF or TSV formats). It includes sanitization, validation and annotation (see below). The TGex annotation server is optimized to rapidly annotate VCF files in under 5 min per whole Exome using proprietary acceleration technologies, and is scalable for large installations.

***TGex Reporting server*** – This service manages report templates for customers and generates patient reports on demand or automatically. Importantly, this server allows each lab to build its own customized reports, which may include any information from the VCF, its annotations, data from the GeneCards knowledgebase, and/or customer proprietary data.

Minimum requirements for using the TGex Web Client are:

- A modern browser (Chrome, FireFox, Safari or Edge)
- An Intel i5, i7 or i9 7th generation or newer processor
- At least 4GB of RAM
- An internet or intranet connection of at least 10Mbit

## Results

TGex is a novel patient-driven web platform for management of clinical genetic tests. It includes annotation, filtering, analysis and interpretation of clinical genetics data. TGex serves as a holistic solution for clinical genetics workflow integration, including management, analysis and reporting of genetic tests, starting from uploading VCF files and going all the way to report generation.

### Clinical genetics workflow with TGex

**Fig. 2:**
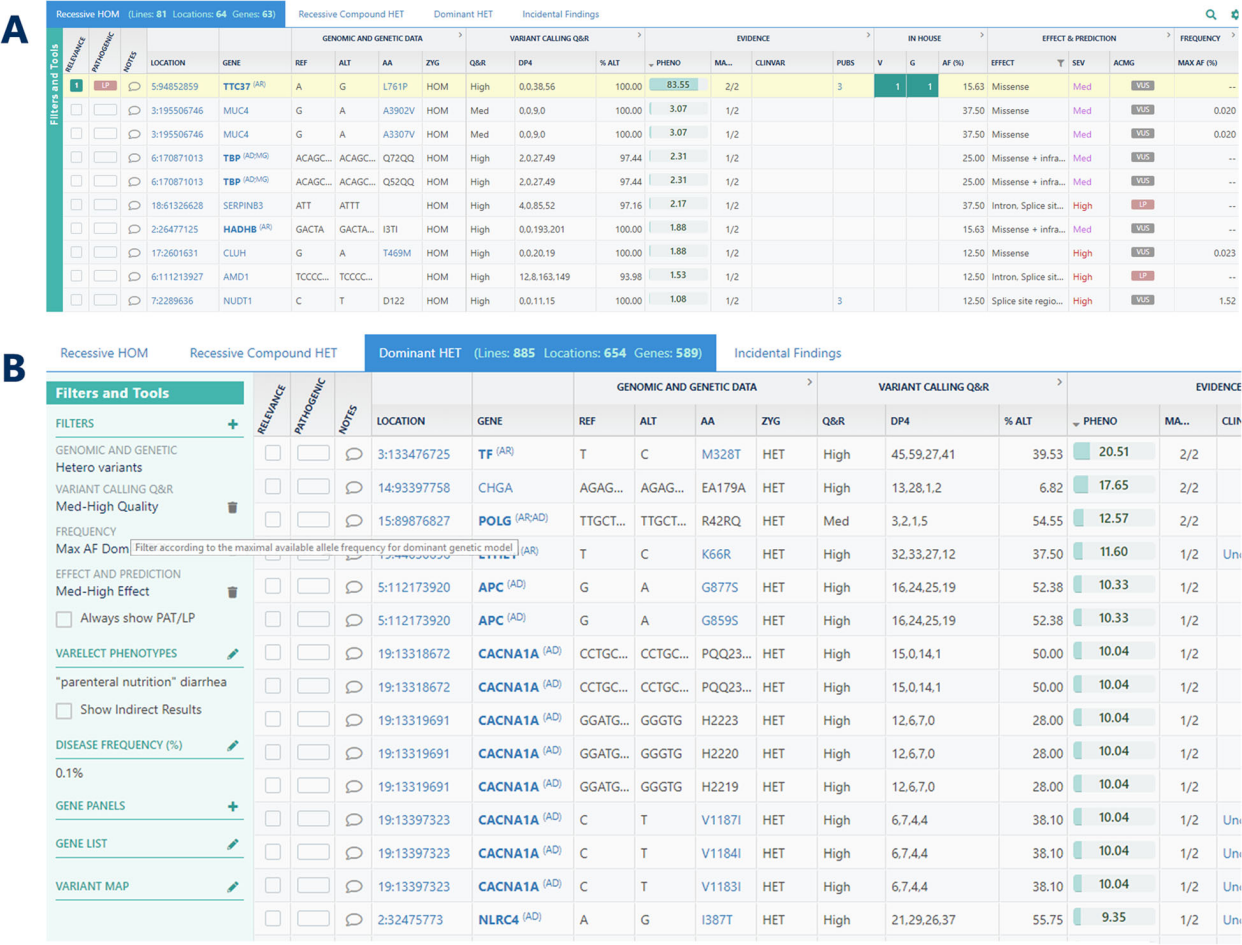
The TGex analysis screen (SNVs). The example shown here (and in Fig. 3) is a case of non-syndromic congenital diarrhea [54]. Following WES, the variant with the strongest phenotype implication for “diarrhea” was within *TTC37* (L761P), a known gene for trichohepatoenteric syndrome. The discovery of this novel homozygous damaging missense variant was significant for providing an effective diagnosis for a misdiagnosed case. **a** The main analysis screen is designed to optimally provide the analyst with information and user-interface options. The main analysis area is divided into dedicated tabs for each genetic model used for the analysis, and an additional tab for incidental findings. Each tab is an interactive table where each row represents a variant, and each column depicts a particular variant attribute. The attributes are divided into 7 categories, each category is collapsed by default, showing a subset of critical attributes, with an option to expand. Each column has two interactive functionalities – sorting (by a click on the header) and filtering (clicking on the filter icon to the right). **b** The Filters and Tools pane summarizes all applied filters for a specific tab in a given analysis. Via this pane, or alternatively via each of the attribute columns in the main analysis screen, filters can be easily added, edited or removed while reviewing the variants. All applied filters are also documented in the Methods section of the final report. In addition to the column filters described above, the pane includes advanced filter options, including predefined gene panels, manually entered gene list filters, VarElect terms used for phenotype prioritization, and Disease frequency used for the allele frequency filter

In this section we describe the clinical genetics workflow within TGex, focusing on identification of rare germline genetic variants, one of the variety of types of analyses that can be performed using TGex (Table 2). The platform is comprised of three main components. The first is the management module, represented by the TGex dashboard, where the user can easily review and access current analyses, or create a new case by uploading a new VCF together with all relevant patient details (Additional file 1: Fig. S1). The second and main module is the analysis component. Following the creation of a new case in TGex, the uploaded VCF file goes through the annotation process described in the Implementation section above. The result is a fully annotated table of variants to be analyzed via the main analysis screen (Fig. 2). The third module is the reporting engine, which collects all relevant patient information, the clinical details, the samples, and most importantly the selected variants and accompanying genetic, biomedical and molecular information from the GeneCards knowledgebase, and consolidates them into a fully automated, comprehensive and customizable report (Additional File 2).

**Table 2:** Examples of protocols in TGex

Analysis Type (Protocol)	Description	Main Sample	Associated Samples
Single Sample Exome	Rare genetic disorders	Proband	N/A
Trio Exome	Rare genetic disorders	Proband	Mother; Father
Tumor Biopsy	Cancer genetics	Tumor biopsy	Matched germline
Carrier Screening	Mendelian disorders	Virtual offspring	Mother; Father
Health Screening	Cancer risk assessment	Patient	N/A
PGx	PharmacoGenomics	Patient	N/A

#### The annotation pipeline

The general flow of clinical genetic tests starts from getting the full list of genetic variants, whether by genotyping specific positions in the genome or, more commonly nowadays, by sequencing large regions in the human genome (e.g. gene panels or Exomes). Recently, even full genomes using NGS and additional technologies are provided. Handling thousands to millions of variants with the aim of identifying the single or perhaps a few mutations that are causal for specific symptoms, clinical conditions, or genetic disorders requires four main steps – annotation, filtering, analysis and interpretation.

Once uploaded, the VCF goes through the annotation pipeline of TGex (Fig. 1), which enriches each variant with dozens of pieces of information using various available data sources and software tools (see also Table 1 and Implementation). These annotations are grouped into 7 categories:

1. **Genomic and Genetic Data**: genomic location, affected gene, reference allele, alternative allele, amino acid change and genotype (zygosity).
2. **Variant Calling Quality and Reliability (Q&R)**: combined quality score, absolute read counts, and the percentage of reads showing the alternative allele.
3. **Evidence**: the VarElect score for the association between the gene and the phenotype terms, the number of matched phenotypes, matching COSMIC [40], CiVIC [39] and ClinVar [38] entries, and publications associated with the variant.
4. **Effect and Prediction**: the effect on the gene, the severity of the effect (combining several prediction algorithms), and the calculated ACMG pathogenicity assignment.
5. **Frequency**: the allele frequency observed in the following control datasets: 1000 Genomes [35], ESP6500 [34], ExAC (including GnomAD) [33] and CONVERGE [37].
6. **In-House**: allele frequency within all of the cases in the account, pointers to previously selected matching variants and genes in all analyzed cases and their interpretations.
7. **Inter-Lab sharing**: allele frequency within all cases in accounts sharing data with this account, pointers to previously selected matching variants and genes in all analyzed cases in the sharing group and their annotations.

#### The user interface

The main analysis screen of TGex is essentially a detailed interactive table, where each row represents a single genomic position with a variation, and each column is populated with the relevant information gathered during the annotation process (Fig. 2a). The user can search or apply filters on any column, and the resulting list or variants can be sorted according to any column, in order to examine the remaining list of variants by their relevance or by their probability of being the causal variants.

One of the important strengths of TGex is the ability to create protocols (Table 2). A protocol in TGex can, if applicable, define which set of genetic models should be analyzed, include combinations of simple or more complex filters on any set of annotation entities, be restricted to certain lists of genes or genomic regions, define a template for a report and the data that should be within it, and much more. The resulting interface is represented by a tab for each genetic model, and a set of filters that are explicitly shown in a collapsible pane on the left side (Fig. 2b). Moreover, each column filter that is applied by the user is also documented in the filter pane. One may apply and/or remove filters during the analysis, and examine the resulting instantly updated list of variants.

To create a consolidated view of the most relevant annotations, the default view hides the additional annotations of each category; users can then choose to expand and review any of the dozens of available annotations. For example, by default, the ‘Effect and Prediction’ category contains 3 columns (Fig. 2a), which includes the effect as provided by SnpEff [30], while the expanded view for this category presents the actual scores from many individual sources and predictions tools, e.g. GERP [48], SIFT [45], PolyPhen-2 [44] and MutationTaster [46]. Importantly, the composition of columns presented in the consolidated view is fully customizable as part of the protocol definitions.

### Workflow examples

#### WES analyses

We start with a common example of a rare congenital genetic disorder case, where the DNA sample of the patient underwent WES. Following a standard primary analysis pipeline, typically BWA-GATK [55], the resulting VCF file lists between 20,000 and 50,000 short nucleotide variants (SNVs) representing substitutions and short insertions/deletions (indels) [35, 56]. The featured protocol in TGex in this case typically includes 4 genetic models – recessive homozygote (HOM), recessive compound heterozygote (HET), dominant HET, and incidental findings (based on ACMG guidelines [20]). The dominant HET genetic model, for instance, automatically applies 4 default filters (Fig. 2b):

1. Genomic and Genetic: Includes HET variants only
2. Variant Calling Q&R: Excludes low reliability variants
3. Frequency: Excludes common variants (using the cutoff set by the user; 0.1% in this case)
4. Effect and Prediction: Excludes variants with low or no predicted effect on the protein function

Even after applying stringent filters, several hundred variants typically remain. The analysis step deals with the challenge of browsing and examining numerous variants, in order to select the best candidates for in-depth review and interpretation. Notably in TGex, the resulting list of variants is sorted according to the VarElect score of the affected gene, which reflects the strength of the association between the gene and the list of user-defined keywords [27], in this case, the list of phenotypes exhibited by the patient. The list of keywords can be defined as part of a customized user protocol, entered manually while initiating a new analysis, and/or modified during the analysis.

**Fig. 3:**
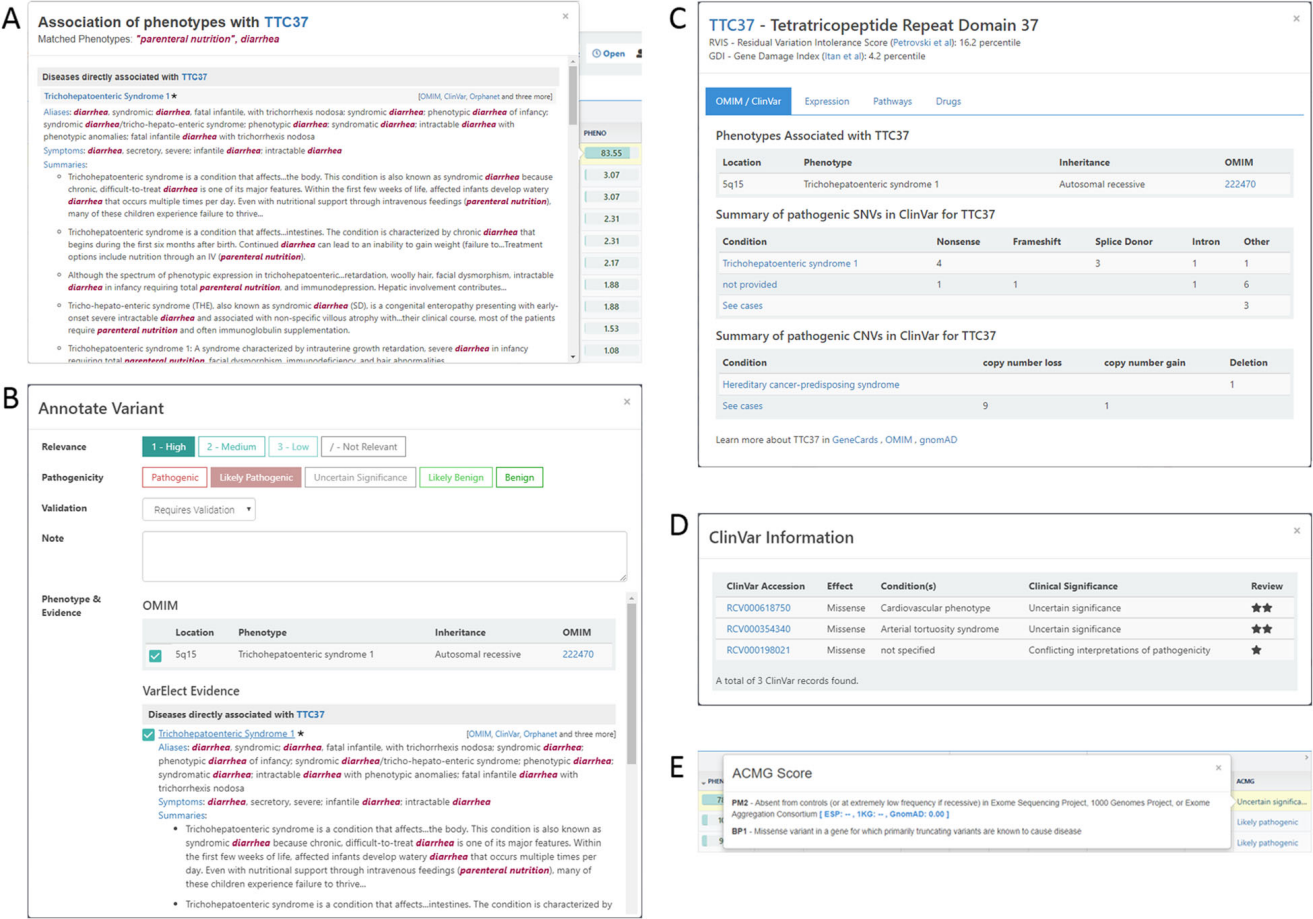
Variant analysis and interpretation. This figure shows several views in TGex providing detailed information and useful links to source data, with the goal to improve and hasten expert variant interpretation. **a** VarElect MiniCards. The extensive gene-phenotype hit-context evidence from the GeneCards knowledgebase is portrayed in the MiniCards. This figure shows selected parts of the MiniCard for the gene *TTC37* and the phenotypes used in the congenital diarrhea case. A list of matched phenotypes is shown in red in the top part, followed by extensive gene-centric evidence for queried phenotype association from various GeneCards sections. This is combined with MalaCards-based evidence, similarly showing queried phenotype associations in diseases associated with the gene *TTC37*, from various MalaCards sections. Search terms are highlighted throughout the text, and links to specific GeneCards/MalaCards webcard positions enable further scrutiny via more detailed evidence exploration within the knowledgebase. **b** Variant and evidence selection. Several types of marks can be defined per candidate variant by the analyst, upon clicking the ‘Annotate variant’ button located to the left of each variant row. This includes relevance (High, Med or Low), the pathogenicity of the variant, and a free text note. Below, information pieces regarding the variant/gene pathogenicity can be selected, based on VarElect MiniCards and OMIM disease records. The selected variants and their annotations are propagated to the report. **c** Gene view. A gene-centric summary for the gene *TTC37*, including associated diseases, mode of inheritance, and pathogenic variants summary, based on OMIM and ClinVar records. **d** ClinVar information – ClinVar records matching a given variant, including the condition and clinical significance. **e** ACMG score – Clinical significance based on the ACMG score. Clicking upon the variant clinical significance value shows a detailed view of the data used for the classification.

The next stage of the recommended analysis workflow is the interpretation and examination of potential candidate variants. The key entity to explore is the phenotype association, hence the default sorting of the candidate variants to review is their VarElect score. Clicking on the VarElect score, one can review a popup containing all of the evidence gathered from various data sources in GeneCards and MalaCards, represented by texts from the knowledgebase, and clearly highlighting matched keywords within their original context (Fig. 3a). The evidence popup also includes links to the relevant sections in the Suite’s websites, together with links to external sites such as PubMed [57], OMIM [43], and others. Subsequently, the user may perform a thorough examination of the candidate variant’s characteristics, such as its reliability (coverage, reads distribution etc.), its predicted effect on the protein (selected prediction tools), its allele frequency in control populations, and clinical information of the gene, which are also consolidated into a single ACMG-guidelines based score (Fig. 3).

Following this close examination of candidate variants, the user can select the most plausible ones and annotate them according to their relevance and pathogenicity, add free text comments, and select the evidence sections provided by VarElect to be integrated into the report (Fig. 3b). The user may then proceed to other genetic models, and end by exploring the variants found in the 59 recommended ACMG incidental findings genes [20].

At this stage, clicking the ‘Report Preview’ button extracts all of the selected variants with their annotation and evidence sections, allowing one to review the current status of the analysis. Once satisfied with the preview, clicking the ‘Generate Report’ button launches the reporting system to generate the final report in the selected format (Word or PDF), and a supplementary Excel table listing all of the variants in each genetic model for future documentation.

#### Whole genome sequence analyses

There are a growing number of large-scale sequencing projects performing WGS [58, 59], and a growing number of hospitals and genetic laboratories that are now transitioning to WGS for interpretation of genetic diseases. WGS can characterize various types of genetic variation in all parts of the genome [19], making the data much more complex for interpretation. A critical example is structural variants (SVs), known to be a major source of pathogenicity [60–62]. The disease-related mechanism of SVs might not involve any overlap with a disease-associated coding gene. Rather, it might act by influencing genes over large distances by altering non-coding functional units such as regulatory elements (promoters and enhancers) and ncRNA genes. Evaluation of the impact of non-coding variants for disease interpretation is a great challenge, and requires novel approaches and increasingly sophisticated software solutions [29].

**Fig. 4:**
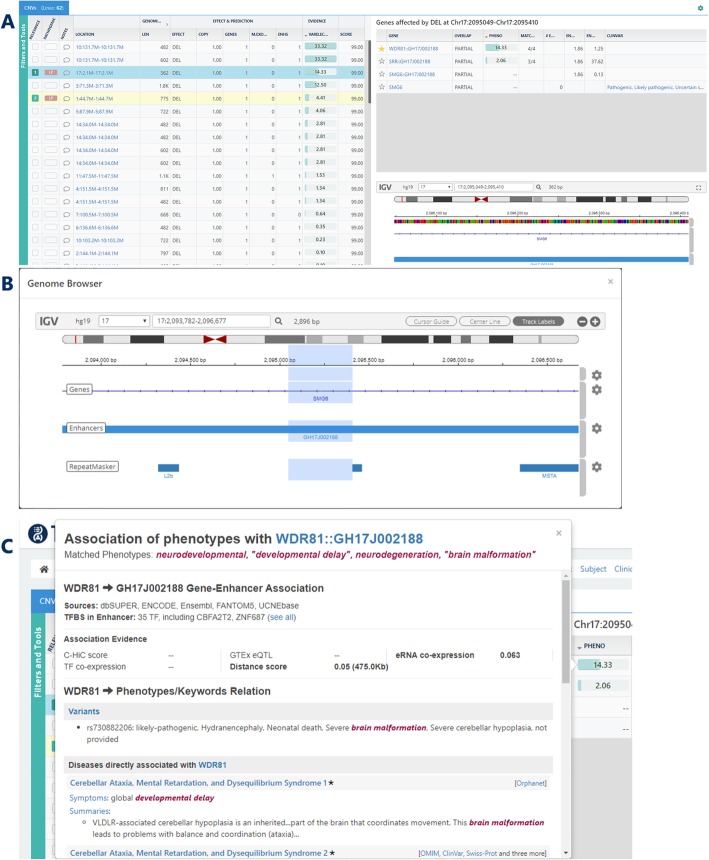
The TGex analysis screen (SVs). SV analysis is exemplified by a list of recurrently mutated regulatory elements discovered in a cohort of patients with neurodevelopmental disorders [63]. The highlighted element overlaps the GH17J002188 GeneHancer, an intronic enhancer of the gene *SMG6*. Remarkably, this enhancer also targets the *WDR81* gene (over ~ 476 kb), with a higher VarElect score for the relevant phenotype (neurodevelopmental, “developmental delay”, etc') than *SMG6*. **a** The main analysis area for SVs is divided into 3 sections, including the main section listing the SV events (left), the detailed event section (top, right) presenting a detailed view of the list of genes and GeneHancer regulatory elements that are affected by the event, and the genomic view section (bottom, right) allowing visual examination of the genomic context of each event. **b** Expanded view of the event genomic context. **c** Clicking on the Phenotype score for a given GeneHancer opens the VarElect MiniCard for the element-gene-phenotype association. At the top part of the MiniCard, evidence describing the GeneHancer and its association with the gene target is detailed. This includes a list of sources for the identification of the element; a list of transcription factors found to have binding sites within the element; a detailed view of the evidence for element-gene associations. Below the GeneHancer details appear the classic gene-phenotype MiniCards as described in Fig. 3. Importantly, the score used for prioritization in the SV module is calculated by combining the GeneHancer confidence score of the element and of the element-gene association, with the classic VarElect gene-phenotype score of the element target gene

For this aim, TGex leverages GeneHancer [26], the GeneCards Suite database of regulatory elements and their gene targets. GeneHancer provides a unique non-redundant and comprehensive genome-wide map of scored ~ 400,000 enhancers and promoters (“GeneHancers”), and their gene associations. The combination of GeneHancer and VarElect enables translating the finding of an SV or SNV variant in a non-coding region into a variant-to-gene-to-phenotype annotation, enabling prioritization of phenotype associations of variant-containing elements via the elements’ gene targets (Fig. 4).

To address the WGS interpretation challenges, we developed a new interface of TGex for WGS data analysis with the focus on complex CNV and SV data. In the GeneHancer-augmented TGex SV module, SVs are mapped to both genes and regulatory elements, followed by VarElect interpretation of the mixed list of genes and enhancers/promoters. VarElect processes GeneHancer element identifiers, performing gene-phenotype prioritization for GeneHancer element gene targets. The phenotype prioritization score in this workflow is calculated by combining the VarElect phenotype score of the element gene target with the GeneHancer element and element-gene association confidence scores.

The CNV/SV analysis screen of TGex is divided into three sections (Fig. 4A). On the left, the user can browse the reported genetic events and use their features, such as the genomic length, the number of affected genes, the copy number, and others, to filter and sort the list of events, quite similarly to the SNV analysis screen. The top right section of the screen lists the genes and the GeneHancers that are affected by the highlighted event and their VarElect score for the input phenotype keywords. The events in the main screen are sorted by default by the highest VarElect score for each event, and therefore increase the probability that the more relevant events appear higher in the main list, markedly reducing the time spent on performing such non-trivial analyses. Clicking on the VarElect phenotype score shows the MiniCards, with evidence for the element-gene-phenotype association (Fig. 4c). The bottom right panel provides an expandable genomic view (Fig. 4b), where the analyst can visually examine the genomic context and assess the relevance of the genetic event in question.

### TGex advantages and comparison with other tools

Platforms for clinical genetics analyses are highly diverse, with numerous academic and commercial tools available, as recently reviewed [29, 64, 65]. Some facets of such workflows are essentially identical across different platforms (e.g. basic variant annotation steps, allele frequency databases, and variant damage prediction). Less standard features, e.g. phenotype-interpretation, must rely on the availability and development of complex databases, and thus often differ significantly among tools. Another aspect which naturally varies among platforms is the user interface capabilities and interactive features. The individual or organizational user would be inclined to select tools that provide a robust workflow with rapid turnover and high diagnostic yield. In this section we summarize the key properties of TGex that make it a leading analysis platform in this respect, supported by literature comparisons and reviews, TGex use-cases, and in-house data.

#### Phenotype-driven interpretation with VarElect

VarElect is our comprehensive phenotype-dependent gene prioritizer [27] used world-wide, with thousands of users and tens of thousands of analyses per year. VarElect takes advantage of the wealth of information in the GeneCards Suite knowledgebase, jointly exploiting the gene- and disease-centric GeneCards [23] and MalaCards [24], as well as the Suite’s free-text Boolean search and scoring capabilities. VarElect thus proficiently matches variant-containing genes to user-submitted disease/symptom keywords. The VarElect algorithm infers direct as well as indirect (“guilt by association”) links between genes and phenotypes. The indirect mode benefits from GeneCards’ diverse gene-to-gene data links, including the broad pathway information in PathCards, the Suite’s unified pathway database that integrates 12 pathway sources [66]. In addition to scoring genes against user-defined keywords, VarElect provides extensive gene-phenotype evidence (via the “MiniCards”), with such evidence hyperlinked to source databases.

VarElect was shown to outperform four other tools (Phenolyzer [67], Exomiser [68], IVA [69] and Phevor [70]) in the original VarElect paper [27]. More recently, Tomar et al. [28] compared the performance of three gene prioritization tools – VarElect, Phenolyzer [67] and OMIMExplorer [71] on a collection of 50 cases, for which the disease causing gene had already been resolved, and on a simulated real life scenario of having only partial phenotype lists. The authors reported that VarElect outperformed both OMIMExplorer and Phenolyzer. Moreover, when omitting critical keywords used for gene ranking, VarElect remained practically unaffected, while both competing tools showed a marked reduction in performance [28].

#### Phenotype prioritization of non-coding variants

To address one of the critical challenges in the analysis of WGS, we developed novel databases and approaches paving the way to interpretation of non-coding variants (see ‘Whole genome sequence analyses’ section above). With GeneHancer, the regulatory element database, and the inclusive collection of ncRNA genes in GeneCards, TGex greatly expands the genomic scope of phenotype-driven analysis, from the commonly used 2% (exome) to ~ 20%. This is a key prospect in the exploitation of WGS to increase the diagnostic yield.

#### Addressing a wide spectrum of genetic analyses

TGex is designed to judiciously leverage the vast GeneCards Suite knowledgebase to address various clinical genetics requirements and workflows. TGex was shown to quickly identify causal mutations of rare disease cases, both when the causal variant was previously documented [72], and in the discovery of novel mutations through prioritization of potential variants of uncertain significance (VOUSes) [73]. Even for patients diagnosed with a known genetic disorder and an identified mutation, TGex has been used to examine phenotypic variability and identify modifier mutations and genes on top of established findings [74]. TGex was also useful in the analysis of novel disorders and syndromes, establishing the evidence for the clinical validity of the association between genes and emerging disorders [75, 76], and in discovery of predisposition variants to complex diseases (e.g. Parkinson) [77], as well as hereditary cancer (see Clinical use cases section below) and molecular profiling of tumor biopsies [78].

#### Robustness and standardization of analysis

Platforms like TGex support performing analyses under robust, consistent, reproducible and standardized conditions, compliant with community best practices recommendations such as HIPAA standards and to ACMG guidelines.

Accounts using TGex benefit from having all analyses stored in a structured database, allowing the organization to benefit from analyzing the case statistics, workflows, bottlenecks, disease and variant trends, etc., as shown in the ‘Large scale account’ section below.

#### In-house allele frequency

TGex handles thousands of samples in specific accounts or group of collaborating accounts and automatically calculates the ‘in-house’ allele frequency which can be crucial for variant selection especially in highly specific ethnic groups. The user interface also includes and highlights the former annotations and interpretations as entered by the analysts, assisting in applying the accumulated in-house knowledge to new cases.

#### True end-to-end all-in-one platform

TGex provides a complete workflow, starting from a VCF file, performing the analysis and interpretation accompanied with evidence scrutinizing, and concluding the findings in the report, all via a user-friendly interface. The immediate consequences are the high diagnostic yield and the fast and cost-effective analysis with the intuitive interface encompassing a broad knowledgebase, optimizing the time it takes an analyst to interpret the data. Together, these features have the potential to markedly increase the volume of cases to be analyzed in large organizations.

Suwinski et al. [79] reviewed how application of biological databases and bioinformatics tools can address the bottleneck in clinical genetics data processing and analysis. Focusing on four currently available web-based interface platforms that include clinical prioritization of variants in VCF files, they conclude that in terms of innovation, depth of knowledge and the ease of generating clinical reports, TGex is the top scorer and is by far the most clinician-friendly WES analysis pipeline and reporting platform [79].

### Clinical use cases

In the past few years, TGex and VarElect have been widely adopted for clinical genetics analysis in various academic institutions, genetic medical centers and hospitals world-wide, with usage volumes ranging from research groups focusing on a handful of patients to genetic centers routinely analyzing hundreds of cases per month [54, 72–78, 80–95]. Our interactions with a diversity of clinical genetics users impel us to deliver frequent community-driven improvements. In this section, we describe three representative studies that illustrate recent use of TGex in different clinical genetics contexts.

#### Cerebral creatine deficiency syndrome-1

Cerebral creatine deficiency syndrome-1 (CCDS1, MIM:300352 [43], MalaCards ID:CRB151 [24]) is an X-linked disorder of creatine transport characterized by mental retardation, severe speech delay, behavioral abnormalities and seizures. Defects in the creatine transporter gene *SLC6A8* have been reported to cause CCDS1 [96]. A 5 year old male patient from a Chinese family was referred for genetic evaluation of development and speech delay and intellectual disabilities at the genetic counselling clinic in the Shenzen Maternal and Child Healthcare Hospital (China) [73]. Following targeted exome sequencing and data analysis with TGex, a novel candidate missense variant, c.1181C > A (p.Thr394Lys) in the *SLC6A8* gene (NM_005629.3) was identified, with high probability as a candidate mutation. Sanger sequencing validation confirmed that the father was not a carrier; the mutation was inherited from the heterozygous carrier mother, and also to the hemizygous similarly affected brother. The diagnosis was further confirmed by biochemical measurements, as well as by brain magnetic resonance spectroscopy. The proband’s mother became pregnant with a 3rd sibling, for whom Sanger sequencing showed a negative result for this variant.

As concluded by the authors [73], this case shows that “The combination of targeted exome sequencing with systematic clinical evaluation of patients used in suspected genetic disorders may improve diagnostic yield, assist in the medical care of patients and offer genetic counseling and prenatal diagnosis for family members.”

#### Large scale account

One remarkable example of extensive use of TGex in a large organization is provided at the Maternal and Child Health Hospital of Guangxi Zhuang Autonomous Region, China. The clinical genetics team at Guangxi has analyzed with TGex more than 3500 samples since 2017. Generating summary statistics and usage trend analyses, we focus on WES analyses of ~ 1300 singletons, and ~ 200 trios (proband and parents). We consider the ‘High’ or ‘Medium’ variant relevance marks, as submitted by the analysts, as an acceptable proxy for a resolved case. We note that the percentage of cases with marked candidates in TGex is comparable to the over-all diagnostic yield as documented by the Guangxi team: about 42% for proband only, and up to 50% in the trio cases (48 and 55% according to TGex relevance marks, respectively).

Notably, since TGex uses keywords for the analysis of rare genetic disorders, we were able to examine the nature of the keyword search as entered by the analyst in each case, and compare between classes of clinical symptoms that were used for the exome analyses. The four main classes of phenotypes (with minor overlap between them) are “Growth Retardation”, “Developmental Delay”, “Epilepsy” and “Genitalia symptoms”. Table 3 summarizes the diagnostic yield in each phenotype class (using the aforementioned approximation marks), showing a much higher yield for the first three classes (~ 60%) while only 23% of the “Genitalia symptoms” cases were resolved. The highest yield in the “Epilepsy” cases could be attributed to the broader clinical genetics knowledge that was gathered during the last decade with hundreds of epilepsy-associated genes and validated mutations. This is in line with previous studies also showing that the diagnostic yield significantly varies among diseases, a phenomenon that might be related to a combination of several factors, including the degree of phenotype complexity, the depth of biomedical knowledge regarding the known causative genes of the specific disease, and others [12, 97, 98].

**Table 3:** Comparison between phenotype classes in Guangxi Maternal Hospital

Phenotype class	Common associated keywords	Total cases	Resolved cases	% Resolved
Growth Retardation	Short stature	107	66	61.7
Developmental Delay	Mental retardation, Delayed speech, Motor delay	174	101	58.0
Epilepsy	Seizures, Convulsion, Spasm	191	121	63.4
Genitalia symptoms	Scrotum, Micropenis, Hypogonadism, Hypospadia	131	30	22.9

To compare between phenotype classes, highly abundant keywords in all of the cases of the account were selected. Those keywords were grouped into four main classes of phenotypes, and the statistics for all of the cases of each phenotype class were calculated (with minor case overlap between classes)

In order to examine the multi-year resolution rate, we compared the work done in 2017 and 2018 (regardless of the search keywords). We observe a significant increase in the number of cases with selected candidate variants, from ~ 42% in 2017 (599 cases) to ~ 65% in 2018 (552 cases), which might be explained by several possible reasons:

- The improved accumulation of clinical genetics data in the source databases integrated within the constantly updated GeneCards Suite knowledgebase.
- Improved adaptation of TGex within the organization and deeper experience of the analysts.
- Accumulation of data in the highly specific in-house database, which assists in variant filtering according to in-house allele frequency.

To summarize, a strong advantage of using a platform in clinical genetics practice, is the standardization of methods and protocols, allowing for simple generation of statistics related to all analyzed samples and cases. Moreover, it will enable automatic re-annotation and re-analysis of unresolved cases, and highlighting the ones worth re-examining due to novel information specifically associated with each case.

#### WGS for Hamartomatous polyposis syndromes

In the last decade, genetic tests of hereditary cancer has rapidly progressed from genotyping germline mutations by single gene Sanger sequencing or mutation panels to large scale sequencing of germline multi-gene panels and WES for diagnostic and prognostic applications [95, 99]. These complex genetic tests can detect more pathogenic genetic alterations, thus enabling better treatment decisions and personally tailored long-term surveillance for mutation carriers in the family. Gastrointestinal (GI) polyps and cancer have a very strong genetic component, with known genes that could be screened in high-risk families. Up to 10% of colorectal cancer (CRC) cases occur due to hereditary genetic syndromes, with even higher numbers for early-onset cases [100, 101]. Rare pathogenic mutations and common genetic variants contribute to personal and familial CRC risk.

In a cohort (with 74 patients in 52 families) presenting with hamartomatous polyposis phenotype with corresponding family history, at the Tel-Aviv Medical Center, the GI team conducted a comprehensive mutational search. Screening, with either cancer multi-gene panels or Sanger sequencing of suspected mutated genes, identified causal mutations in only ~ 50% of the families. The team selected 5 probands in which the genetic evaluation produced no significant findings, and performed a much wider search using WGS in TGex, envisioning that some mutations would not necessarily be SNVs but rather CNVs and SVs which could be identified by whole genome analysis. For each sample in this set, variant calling for SNVs and SVs was conducted, and both variant files were uploaded to TGex to a combined SNV/SV protocol. The analysis of these cases using the relevant keywords was simple and efficient, quickly pointing out the most relevant candidate variants, whether SNVs or SVs.

Remarkably, the genetic culprit was detected in all 5 cases following rapid analysis with TGex, presenting the subsequently validated causal events at the highest ranks, out of thousands of called variants. This included a loss-of-function SNV in *BMPR1A* (for this proband the suspected gene was *SMAD4* for which Sanger sequencing produced no clinically significant finding) and three SV events – two distinct cases of inversions, one affecting *BMPR1A* and the other affecting *STK11*, and a deletion in *BMPR1A* identified in two unrelated probands of a common ethnic origin, a possible founder mutation. *BMPR1A* (Bone Morphogenetic Protein Receptor Type 1A) is a cancer predisposing gene, related to polyposis, e.g. Juvenile polyposis syndrome (MIM:174900 [43], MalaCards ID:JVN014 [24]), an autosomal dominant GI cancer. All *BMPR1A* events were validated among the probands and affected family members by PCR and MLPA.

These results, although based on a small number of cases with a unique clinical phenotype, imply the potential of WGS, specifically with using the accurate and simplified TGex CNV/SV analysis, to markedly increase the diagnostic yield of genetic tests, leading the way to accurate genetic diagnosis in a timely and cost effective manner.

### Future perspectives

The near future holds great promise for clinical genetics. Recent advances have made significant impact, however, analysis and interpretation of genome variation still remain challenging. Clinical genetics platforms like TGex are expected to be continually augmented with ever-growing variant- and gene-disease phenotype association knowledge, stronger variant frequency catalogs, and improved algorithms. This will be complemented with essential efforts aiming at comprehensive variant detection of the whole gamut of variant classes (e.g. mobile elements, tandem repeats), and at improved ability to interpret non-coding variants within functional genomic regions (e.g. regulatory elements and ncRNA genes).

The GeneCards Suite is a leading biomedical knowledgebase, serving as a solid foundation for the clinical genetics variant interpretation capacities of TGex. Our effort to characterize the genomic “dark matter” arena of non-coding regions is focused on enhancing the non-coding variants interpretation capacities within TGex. With the aim of continuing our innovative development towards improved variant interpretation, the GeneCards Suite future effort will constitute significantly enhanced annotation of genome-wide functional non-coding elements, so as to allow TGex to find direct and indirect phenotype associations of those regions.

## Conclusions

TGex is a powerful tool for the annotation, analysis and prioritization of coding and non-coding genomic variants. It provides access to an extensive knowledgebase of genomic annotations, with intuitive and flexible configuration options, allowing quick adaptation, and addressing various workflow requirements, simplifying and accelerating variant interpretation. TGex can be used in the various scenarios typically found in clinical organizations, e.g. by an analyst who creates an intermediate report for the clinical geneticist, or by a genetics team generating the final clinical report, based on a gene panel, Exome, or whole genome analysis. For all of these situations, TGex has a great potential to markedly reduce turn-around time by enabling methodical and faster analysis for primary analysts, followed by efficient review by geneticists. We have shown how the unique combination of TGex’s strengths are increasingly useful for clinicians and researchers, and expect TGex to open new vistas for WGS in clinical genetics.

## Availability and requirements

**Project name**: TGex

**Project home page**: https://tgex-app.genecards.org/ or https://tgex-app.genecards.cn (China)

**Operating system(s)**: Platform independent (any operating system)

**Programming language**: .NET (back-end) and HTML/JavaScript (front-end)

**Other requirements**: A modern browser and processor; an internet or intranet connection.

**License**: Free academic research use

**Any restrictions to use by non-academics**: License required

## Supplementary information

**Additional file 1: Figure S1.** The TGex dashboard - account management module.

**Additional file 2.** TGex report for the trichohepatoenteric syndrome Demo example

## Data Availability

The SNV example (Fig. 2, Fig. 3) is available for each new TGex account as a demo case. The latest TGex documentation is available at http://tgex.genecards.org/user-guide/

## Abbreviations

API: Application Programming Interface
CCDS1: Cerebral Creatine Deficiency Syndrome-1
CRC: Colorectal Cancer
EHR: Electronic Health Record
GDPR: General Data Protection Regulation
GI: GastroIntestinal
HET: Heterozygote
HIPAA: Health Insurance Portability and Accountability Act
HOM: Homozygote
indels: Insertions/deletions
LIMS: Laboratory Information Management Systems
LMS: LifeMap Sciences
ncRNA: Non-coding RNA
NGS: Next Generation Sequencing
Q&R: Quality and Reliability
SNVs: Short Nucleotide Variants
SVs: Structural Variants
TGex: Translational Genomics expert
VCF: Variant Call Format
VOUS: Variant Of Uncertain Significance
WES: Whole Exome Sequencing
WGS: Whole Genome Sequencing
WIS: Weizmann Institute of Science
