# Supplementary material for: Genome analysis and knowledge-driven variant interpretation with TGex
Source: BMC Med Genomics. 2019 Dec 30;12:200. doi: 10.1186/s12920-019-0647-8 (PMC6937949; doi:10.1186/s12920-019-0647-8)
Supplement: Supplementary file 1 — Additional file 1: Figure S1. The TGex dashboard - account management module. [file 12920_2019_647_MOESM1_ESM.pdf]

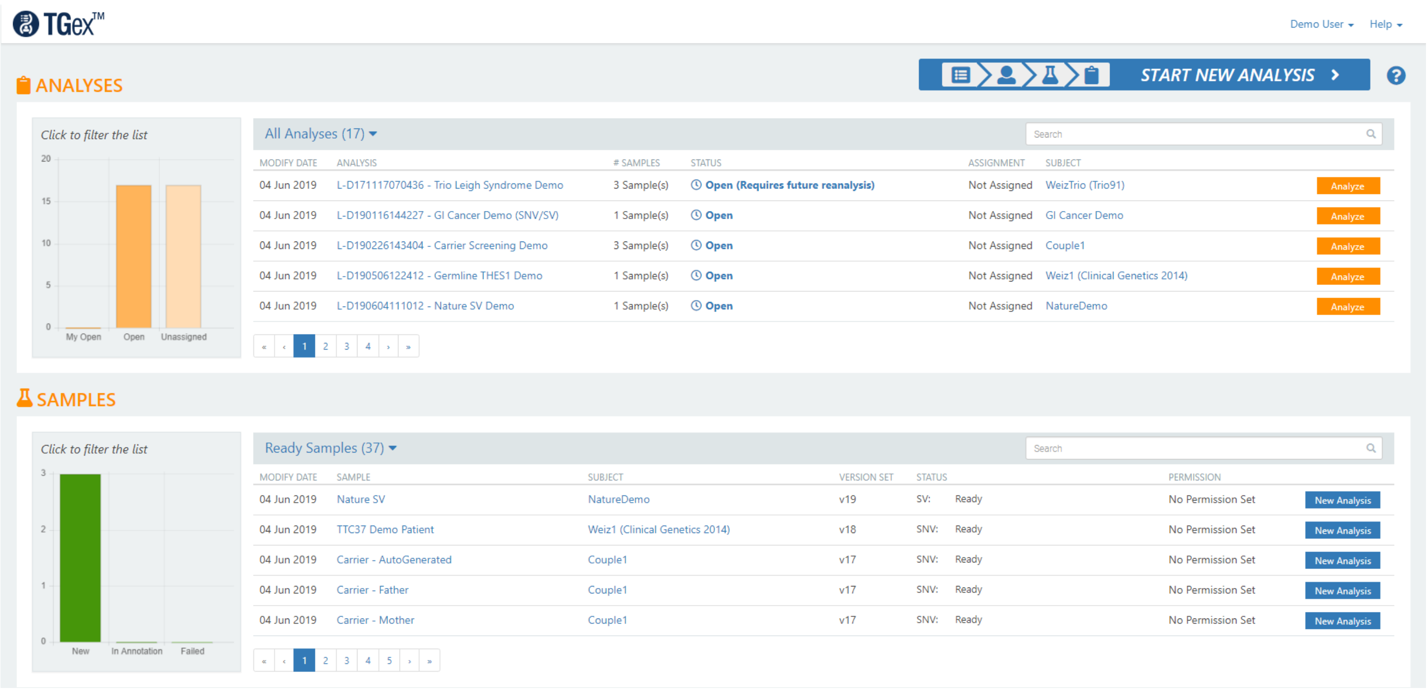


*Supplementary Figure 1*

**The TGex dashboard - account management module**

The major entities in TGex are subjects, samples and analyses. A subject's attributes encompass general details which are available during the analysis process, and automatically provided in the report. These include: A unique identifier, e.g. the patient identifier in an EHR/LIMS system; Name; Birth date; Gender; Consanguinity, commonly used in cases of rare disease analysis; Population type; Paternal and maternal ancestry; Family history. Each subject might be associated with multiple samples, and each sample might belong to multiple analyses. TGex is subject-centric, hence each analysis contains a single main sample, with the option to be accompanied by several associated samples (for example: the parents in a trio analysis, or the matched germline in a tumor biopsy analysis).

An analysis has additional properties: Serial Number; Name; Owner, the name of the referring clinician; Description, free text dedicated to describe the clinical aspects of the case; Disease frequency, expected frequency of the disease in the population, defining the frequency filter in the analysis (default = 0.1%); Genetic models; Phenotype terms for gene-phenotype interpretation; User-defined gene list filter to be used during analysis.

The Analyses section (top pane) contains two areas: (1) Analyses list (top, right). For each analysis record, the following additional details are shown: Last modification date; Name and identifier; Number of samples used; Analysis status; Assignment; Subject name. Depending on the analysis status, an "Analyze" button (for analyses without a generated report), or a "View" button (for ones with a report), launches the analysis screen or the summary page,

respectively. (2) Summary graph (top, left). This shows a view of the number of analyses that are still pending analysis, or those that are missing samples (and therefore cannot yet be analyzed).

The Samples section (bottom pane) contains two areas: (1) Samples list (bottom, right). For each sample record additional details are shown: Last modification date; Sample Name; Subject Name; Version Set - The version set used to annotate this sample; Sample annotation status (‘Ready’, ‘In Annotation’, ‘Failed’). A "New Analysis" button triggers a new analysis for a sample. (2) Summary graph (bottom, left). This shows a view of unanalyzed samples, split by their status (‘New’/’In Annotation’/’Failed’).
