# Supplementary material for: Genome analysis and knowledge-driven variant interpretation with TGex
Source: BMC Med Genomics. 2019 Dec 30;12:200. doi: 10.1186/s12920-019-0647-8 (PMC6937949; doi:10.1186/s12920-019-0647-8)
Supplement: Supplementary file 2 — Additional file 2. TGex report for the trichohepatoenteric syndrome Demo example [file 12920_2019_647_MOESM2_ESM.pdf]

Sequencing Analysis Report

Patient

Name: **Clinical Genetics 2014**
Date of Birth: **5 Jan 2010**
Gender: **F**
Patient ID: **Weiz1**
Accession # **TTC37 Demo Patient**

Healthcare Provider

LifeMap Demo

Main Specimen(s)

Specimen Type: **GermLine**
Accession Date: **1 Dec 2015**
Draw Date: **1 Nov 2015**
Report Date: **30 Sep 2019**

Clinical Information

A 4-year-old girl in a consanguineous family with no gastrointestinal history. Developed persistent secretory diarrhea at 18 days of age and has been completely dependent on total parenteral nutrition (TPN). Has minor dysmorphic features but no obvious developmental delay.

Phenotypes: **diarrhea, "parenteral nutrition", ftt**

Main findings: variant(s) identified in TTC37

Details about L761P (NM_014639.3) [Recessive HOM] - Relevance: High - Likely Pathogenic

The homozygote A->G substitution at chr5:94852859 is predicted to result in abnormal protein translation of the TTC37 protein at amino acid position 761.

Predicted effect(s) on the protein: Missense

The quality and reliability of the variant calling is High and the severity of the impact on the protein is Med.

This variant was not found in the available control databases (see methods).

Variant Note: Associated with Trichohepatoenteric syndrome 1, which matches phenotypes

Clinical Significance of TTC37

Indications

TRICHOHEPATOENTERIC SYNDROME 1; THES1 [MIM:222470]

Although the spectrum of phenotypic expression in trichohepatoenteric syndrome (THES) is broad, the characteristic features include intrauterine growth retardation, woolly hair, facial dysmorphism, intractable diarrhea in infancy requiring total parenteral nutrition, and immunodepression. Hepatic involvement contributes to the poor prognosis of affected patients (summary by Fabre et al., 2007[1]).

Genetic Heterogeneity of Trichohepatoenteric Syndrome

Trichohepatoenteric syndrome-2 (THES2; 614602) is caused by mutation in the SKIV2L gene (600478) on chromosome 6p21.

Reference(s):

1. Intractable diarrhea with 'phenotypic anomalies' and tricho-hepato-enteric syndrome: two names for the same disorder. [PMID: 17318842]

Evidence from GeneCards Suite Knowledgebase

Diseases directly associated with TTC37

Trichohepatoenteric Syndrome 1 [OMIM, ClinVar, Swiss-Prot and four more]

Aliases:

diarrhea, syndromic; ***diarrhea***, fatal infantile, with trichorrhexis nodosa; syndromic ***diarrhea***; phenotypic ***diarrhea*** of infancy; syndromic ***diarrhea***/tricho-hepato-enteric syndrome; phenotypic ***diarrhea***; syndromatic ***diarrhea***; intractable ***diarrhea*** with phenotypic anomalies; fatal infantile ***diarrhea*** with trichorrhexis nodosa

Symptoms:

diarrhea, secretory, severe; infantile ***diarrhea***; intractable ***diarrhea***

Summaries:

- Tricho-hepato-enteric syndrome (THE), also known as syndromic or phenotypic ***diarrhea***, is an extremely...
- Trichohepatoenteric syndrome is a condition that affects...the body. This condition is also known as syndromic ***diarrhea*** because chronic, difficult-to-treat ***diarrhea*** is one of its major features. Within the first few weeks of life, affected infants develop watery ***diarrhea*** that occurs multiple times per day. Even with nutritional...
- Trichohepatoenteric syndrome is a condition that affects...intestines. The condition is characterized by chronic ***diarrhea*** that begins during the first six months after birth. Continued ***diarrhea*** can lead to an inability to gain weight (failure to...
- Although the spectrum of phenotypic expression in trichohepatoenteric...retardation, woolly hair, facial dysmorphism, intractable ***diarrhea*** in infancy requiring total parenteral nutrition, and...
- Trichohepatoenteric syndrome 1: A syndrome characterized by intrauterine growth retardation, severe ***diarrhea*** in infancy requiring total parenteral nutrition, facial...

Publications

- Identifying Mutations of the Tetratricopeptide Repeat...Domain 37 (TTC37) Gene in Infants With Intractable ***Diarrhea*** and a Comparison of Asian and Non-Asian Phenotype... (PMID: 26945392)
  Abstract:

Syndromic ***diarrhea***/tricho-hepato-enteric syndrome (SD/THE) is a rare...immunodeficiency diseases (PIDs). Neonates with intractable ***diarrhea*** underwent immunologic assessments including immunoglobulin...nonconsanguineous parents, suffered from intractable ***diarrhea***...

Mesh Term:

Diarrhea, Infantile

- Tricho-hepato-enteric syndrome (THE-S): two cases and review of the literature. (PMID: 25976726)

  Abstract:

  Tricho-hepato-enteric syndrome (THE-S) is characterized by severe infantile ***diarrhea***, failure to thrive, dysmorphism, woolly hair, and...sequencing (WES). Both cases presented with chronic ***diarrhea***, failure to thrive, and recurrent infections. Case... • Tricho-Hepato-Enteric syndrome (THE-S) is characterized by severe infantile ***diarrhea***, failure to thrive, dysmorphism, woolly hair, and...

  Mesh Term:

  Diarrhea, Infantile

- Novel mutations in SKIV2L and TTC37 genes in Malaysian children with trichohepatoenteric syndrome. (PMID: 27050310)

  Abstract:

  Trichohepatoenteric syndrome (THES) is a rare autosomal...disorder that is classically associated with intractable ***diarrhea*** with an onset within the first few months of life...

  Mesh Term:

  Diarrhea, Infantile

Methods

Data sources used for variant annotation

Version Set v18

Civic	2019-03	ClinVar	v2019_03	Cnv	2019-02	Converge	2017-04
Cosmic	v87	DbNsfp	v2.9.3a	DbscSNV	2017-04	DbSnp	v151
ExAc	v2.0.2 (gnomAD)	GenomeRef	hg19	MasterMind	2018-11	MitoMap	2017-01
RefSeq	RefSeq-hg19	Rmsk	hg19	SnpEff	v4_3		

Protocol and genetic models

Protocol Germline - Single Sample

Genetic Model Recessive HOM, Recessive Compound HET, Dominant HET, Secondary Findings

Summary of variants in the supplementary Excel

Recessive HOM **242** Locations in **228** Genes

Applied filters - Homo variants, Max AF Rec filter, Med-High Effect, Med-High Quality

Recessive Compound HET **237** Locations in **83** Genes

Applied filters - CompHet genes, Hetero variants, Max AF Rec filter, Med-High Effect, Med-High Quality

Dominant HET **571** Locations in **503** Genes

Applied filters - Hetero variants, Max AF Dom filter, Med-High Effect, Med-High Quality

Secondary Findings **216** Locations in **43** Genes

Applied filters - ACMG, Med-High Quality

* In cases where Max AF filter was applied the cut off used was 0.1%

Gene panels and variant prioritization

Keywords used for VarElect Scoring diarrhea, "parenteral nutrition", ftt

Disclaimer: We continually perform ongoing evaluations of variant classifications. In certain cases, healthcare providers may be contacted for more information or to arrange family testing to aid in variant classification. When new evidence about a variant is identified and determined to result in significance and management change, that information will automatically be made available to the healthcare provider through an amended report.
